# Supplementary material for: Treatment of hypertension by increasing impaired endothelial TRPV4‐KCa2.3 interaction
Source: EMBO Mol Med. 2017 Sep 12;9(11):1491–503. doi: 10.15252/emmm.201707725 (PMC5666316; doi:10.15252/emmm.201707725)
Supplement: Supplementary file 1 — Appendix [file EMMM-9-1491-s001.pdf]

Treatment of hypertension by increasing impaired endothelial TRPV4-KCa2.3 interaction

Authors: Dongxu He[1,#], Qiongxi Pan[1,#], Zhen Chen[1,#], Chunyuan Sun[1,#], Peng Zhang[1], Aiqin Mao[1], Yaodan Zhu[1], Hongjuan Li[1], Chunxiao Lu[1], Mingxu Xie[1], Yin Zhou[1], Daoming Shen[1], Chunlei Tang[1], Zhenyu Yang[2], Jian Jin[1], Xiaoqiang Yao[3], Bernd Nilius[4], Xin Ma[1, *]

APPENDIX

Table of contents

Appendix Fig S1. Specificity of anti-TRPV4, anti-KCa2.3 and anti-flag antibodies.

Appendix Fig S2. Co-Immunoprecipitation (co-IP) of TRPV4, KCa2.3 and caveolin-1 in ECs.

Appendix Fig S3. Patch clamp recording GSK1016790A or CyPPA-stimulated whole cell current.

Appendix Fig S4. EDHF is the dominant component of vasodilation in small arteries.

Appendix Fig S5. Effect of TRPV4 mutation on cellular localization and function of the channel

Appendix Fig S6. Effect of KCa2.3 mutation on cellular localization and function of the channel.

Appendix Fig S7. Interactions between caveolin-1, TRPV4, and KCa2.3 in ECs.

Appendix Fig S8. Effect of M*β*CD on TRPV4-KCa2.3 interaction.

Appendix Fig S9. The proposed binding sites for JNc-440 to TRPV4 and KCa2.3.

Appendix Fig S10. Protective effect of JNc-440.

Appendix Fig S11. JNc-440 has no effect on either (**A**) TRPV4 or (**B**) KCa2.3 channel activity.

Appendix Table S1. Baseline characteristics of Normotensive Control subjects and Hypertensive Patients

Fig. S1

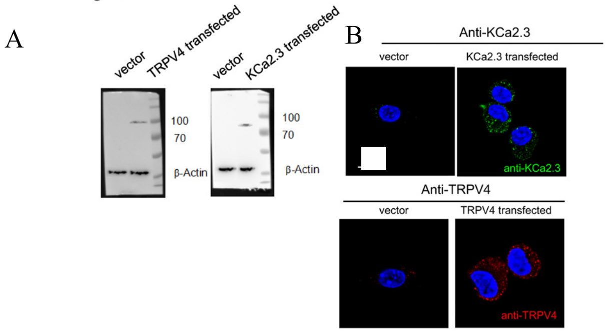

Fig S1 Specificity of anti-TRPV4, anti-K and anti-flag antibodies. TRPV4 or KCa2.3 plasmids were transiently transfected into HEK293 cells, and specificity was validated by western blotting (A) and immunostaining (B). Scale bars, 10 µm.

Fig. S2

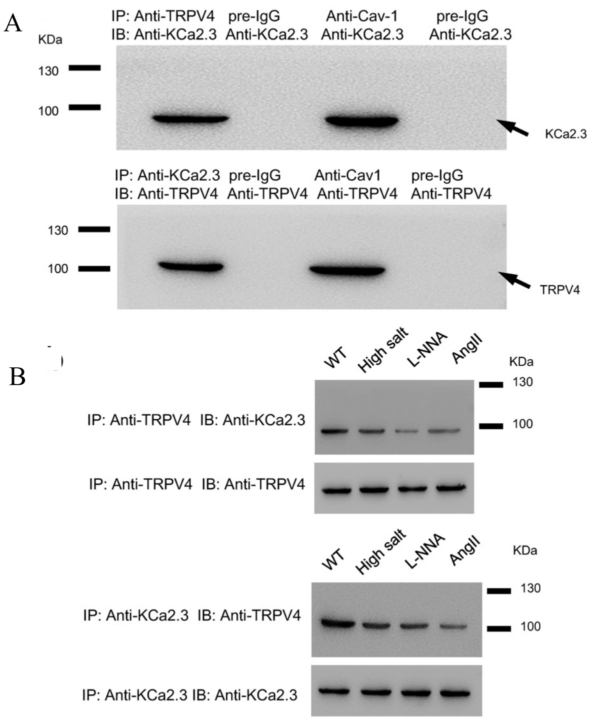

Fig S2 Co-Immunoprecipitation (co-IP) of TRPV4, KCa2.3 and caveolin-1 in ECs. In normotensive mice (A) or mice treated with a high-salt diet, L-NNA, or AngII (B), colocalization of caveolin-1, TRPV4-KCa2.3 interaction was studied by Co-IP.

Fig. S3

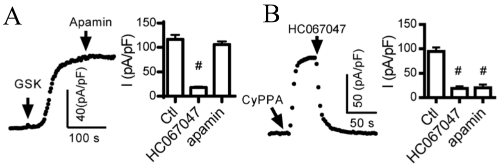

Fig S3 Patch clamp recording of GSK1016790A or CyPPA-stimulated whole cell current at ± 80 mV. **(A)** Effect of apamin on GSK1016790A-stimulated current in primarily cultured mice ECs. **(B)** Effect of HC067047 on CyPPA-stimulated current in primarily cultured mice ECs. Representative traces (left) and summary of data (right) were shown. Data are shown in mean ± SD. #, $P<0.0001$ compared to control by one way ANOVA.

Fig. S4

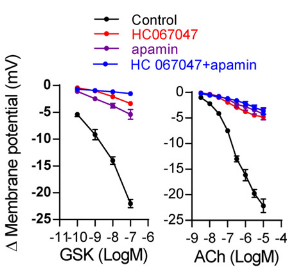

Fig. S4 EDHF is the dominant component of vasodilation in small arteries. **(a)** Effect of apamin and HC067047 on GSK1016790A or ACh -induced hyperpolarization of smooth muscle in isolated mesenteric artery strips. Membrane potentials were measured by sharp microelectrodes impaled from adventitial side. Left: GSK1016790A (GSK, 0.1 nM-0.1 μM). Right: ACh (3 nM-10 μM). Arterial strips were pretreated with HC067047 (10 μM) or apamin (200 nM) or together for 30 min. Data in mean ± SD.

Fig. S5

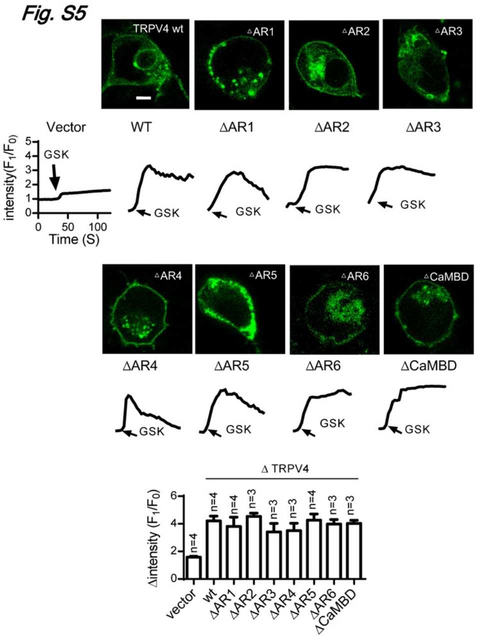

Fig. S5 Effect of TRPV4 mutation on cellular localization and function of the channel. Locations of the channels were analyzed using confocal images, and their function was validated by measurements of Ca^{2+} influx (curves below and summary in the bottom) in response to stimulation with GSK1016790A (GSK), a specific TRPV4 agonist. Scale bars, 5 μm. Data are the mean ± SD.

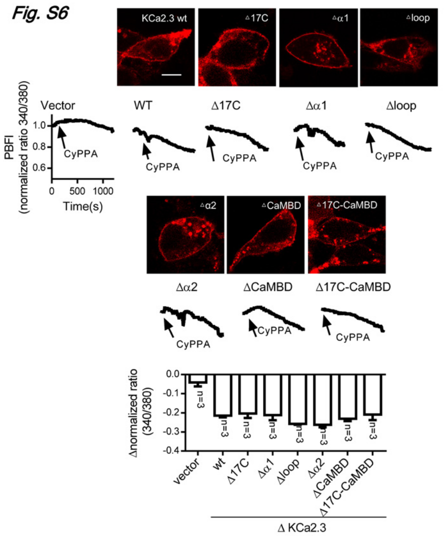

Fig. S6 Effect of KCa2.3 mutation on cellular localization and function of the channel. Locations of the channels were analyzed using confocal images, and their function was validated by measurements of K^+ efflux (curves below and summary in the bottom) in response to stimulation with CyPPA, a specific KCa2.3 agonist. Scale bars, 10 µm. Data are the mean ± SD. Scale bar, 10 µm,

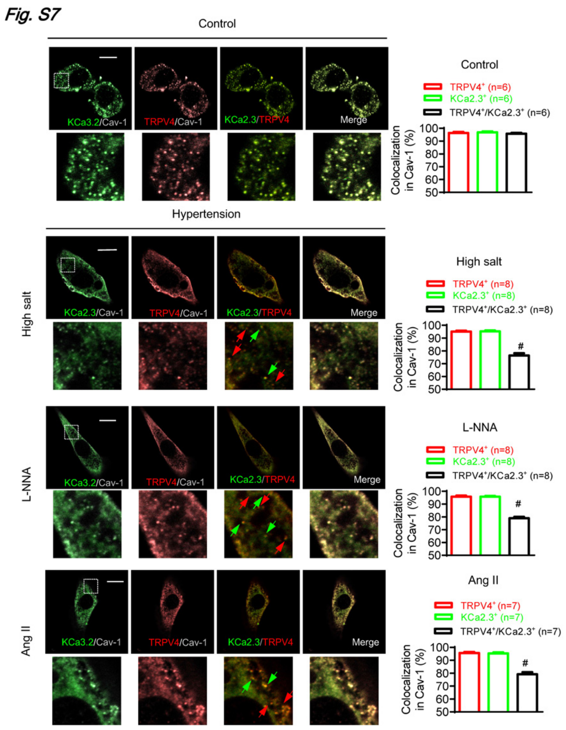

Fig. S7 Interactions between caveolin-1, TRPV4, and KCa2.3 in ECs. In mice treated with a high-salt diet, L-NNA, or AngII, although TRPV4 and KCa2.3 still individually colocalized with caveolin-1, TRPV4-KCa2.3 interaction was reduced compared with in the controls. Representative images and summary data of $TRPV4^+$, $KCa2.3^+$, and $TRPV4^+/KCa2.3^+$ $Cav\text{-}1^+$ signals in ECs from mice treated with a high-salt diet, L-NNA or AngII. Red arrows, TRPV4 signal separated from the $Cav\text{-}1^+$ signal; Green arrows, KCa2.3 signal separated from the $Cav1^+$ signal. Scale bar, 10 µm. Data are the mean ±

SD. $^{\#}p < 0.0001$ compared to controls by one way ANOVA. TRPV4^{+}, SKCa2.3^{+} and Cav-1^{+} indicate positive TRPV4, KCa2.3 and caveolin-1 signal in caveolae.

Fig. S8

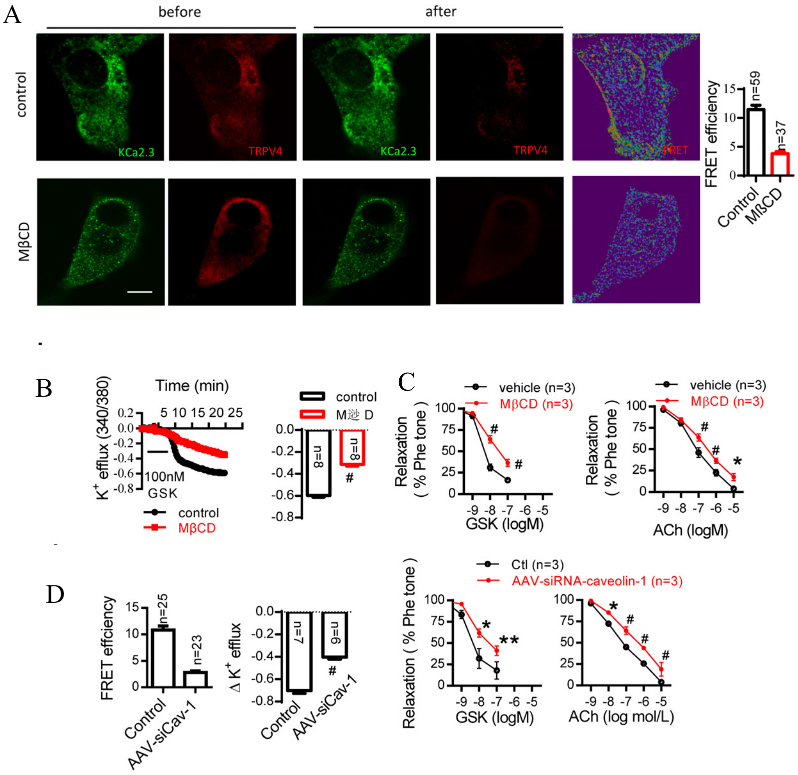

Fig. S8 MβCD decreases (**A**) TRPV4-KCa2.3 interaction *in vitro*, (**B**) GSK1016790A (GSK)-induced K^{+} efflux from primary ECs, #, p<0.0001 by unpaired t test *v.s* control and (**C**) both GSK1016790A-induced and ACh-induced vasodilation in freshly isolated arterial segments. *, p=0.002, # p<0.0001 *v.s* control by two way ANOVA. (**D**) Effect of AAV-siRNA-caveolin-1 (AAV-siCav-1) on TRPV4-KCa2.3 interaction (left) and GSK1016790A-induced K^{+} efflux (2nd panel from left, #, p<0.0001 by unpaired t test *v.s* control) was measured in primary ECs by Immuno-FRET and Fluorescent K^{+} efflux assay respectively. Effect of AAV-siRNA-caveolin-1 on GSK1016790A-induced (3rd panel from left, *, p=0.0001, **, p=0.0015 *v.s* control by two way ANOVA) and ACh-induced vasodilation (right, *, p=0.0005, #, p<0.0001 *v.s* control by two way ANOVA.) was measured in freshly isolated arterial segments by wire myograph. Scale bars, 10 μm. Data are the mean ± SD.

Fig. S9

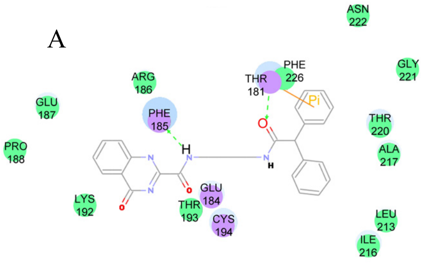

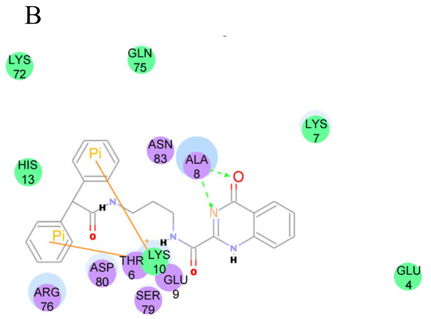

Fig. S9 The proposed binding sites for JNc-440 to TRPV4 and KCa2.3. (**A**) The amino acid residues THR181, PHE226, PHE185, and CHR193 in the TRPV4 molecule were the main sites of interaction between JNc-440 and TRPV4. An amide group in JNc-440 formed a hydrogen bond with PHE185, a carbonyl group formed a hydrogen bond with THR181, and a phenyl group had a Pi interaction with PHE 226; the aromatic rings were buried in the inner hydrophobic pocket. (**B**) The amino acid residues ALA8, THR6, and LYS10 in KCa2.3 were the main sites of interaction between JNc-440 and KCa2.3. Carbonyl and imine groups in JNc-440 formed hydrogen bonds with ALA8, and a diphenyl group had a Pi interaction with THR6 and LYS10, stabilizing the conformation.

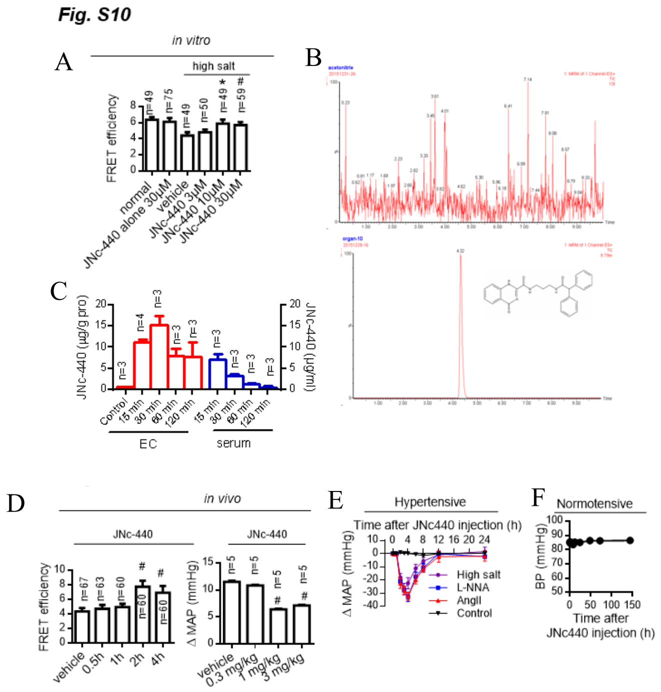

Fig. S10 Protective effect of JNc-440. (**A**) In primary cultured ECs in a high-salt medium, JNc-440 protected TRPV4-KCa2.3 interaction in a dose-dependent manner. *, p=0.00264, #, p=0.0195 *v.s* vehicle by one way ANOVA **(B)** Chromatograms for JNc-440 in plasma samples. Upper panel, blank plasma; Lower panel, blank plasma spiked with JNc-440. (**C**) Concentration of JNc-440 in ECs and serum. JNc-440 (10 mg/kg) was intravenously injected and measured at 15, 30, 60 and 120, min after dosing (n = 5; μg/mL for serum; μg/g for the ECs). **(D)** In mice on a high-salt diet, 1mg/kg JNc-440 protected TRPV4-KCa2.3 interaction in isolated arterial ECs after 2 h after tail injection (left), and had an antihypertensive effect after one week after tail injection (right). #, p<0.0001 *v.s* vehicle by one way

ANOVA. **(E-F)** Time-course of acute blood pressure measurement in high-salt, L-NNA, AngII-treated mice (E) and control (F) after tail injection of 1 mg/kg JNc440. Data are the mean ± SD. $^{\#}p <$ 0.05 compared to vehicle.

Fig. S11

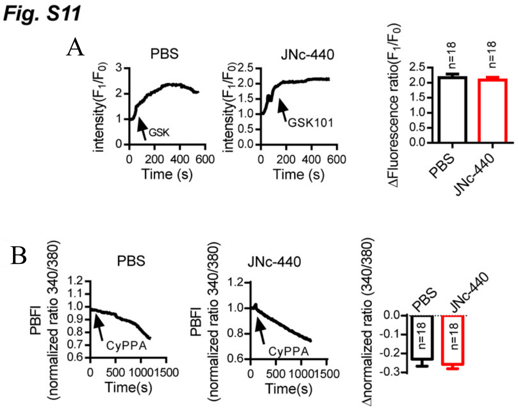

Fig. S11 JNc-440 has no effect on either (**A**) TRPV4 or (**B**) KCa2.3 channel activity. JNc-440 was intravenously injected at 1 mg/kg in normotensive mice, and arterial ECs were isolated. TRPV4 channel function was validated by measuring Ca^{2+} influx with GSK1016790A (GSK). KCa2.3 channel function was validated by measuring K^{+} efflux with CyPPA.

Appendix tables

Table S1 Baseline characteristics of Normotensive Control subjects and Hypertensive Patients

Parameter	Normotensive Controls (n=21)	Hypertensive Patients (n=15)
Gender (M/F)	10/11	8/7
Age (years)	43.3±9.4	51.3±10.7
Systolic blood pressure (mm Hg)	110±8	161±25
Diastolic blood pressure (mm Hg)	67±8	101±17
Heart rate (bpm)	70±8	75±11
Diabetes (%)	2 (9.5)	7 (46.7)
Hyperlipemia (%)	4 (19.0)	10 (66.7)
Atherosclerosis (%)	3 (14.3)	9 (40.0)
Anti-hypertensive drugs (%)	0 (0.0)	10 (66.7)
Femoral artery OD (0.90~1.65 mm)*	9 (42.9)	6 (40.0)
Popliteal artery OD (0.68~1.30 mm)	4 (19.0)	3 (20.0)
Superior Mesenteric artery OD (0.75~1.25 mm)	2 (9.5)	1 (6.7)
Brachial artery OD (0.72~1.45mm)	2 (9.5)	2 (13.3)
Carotid artery OD (0.58~0.96 mm)	4 (19.0)	3 (40.0)

*OD, outer diameter
