# Supplementary figures and images for: Treatment of hypertension by increasing impaired endothelial TRPV4‐KCa2.3 interaction
Source: EMBO Mol Med. 2017 Sep 12;9(11):1491–503. doi: 10.15252/emmm.201707725 (PMC5666316; doi:10.15252/emmm.201707725)

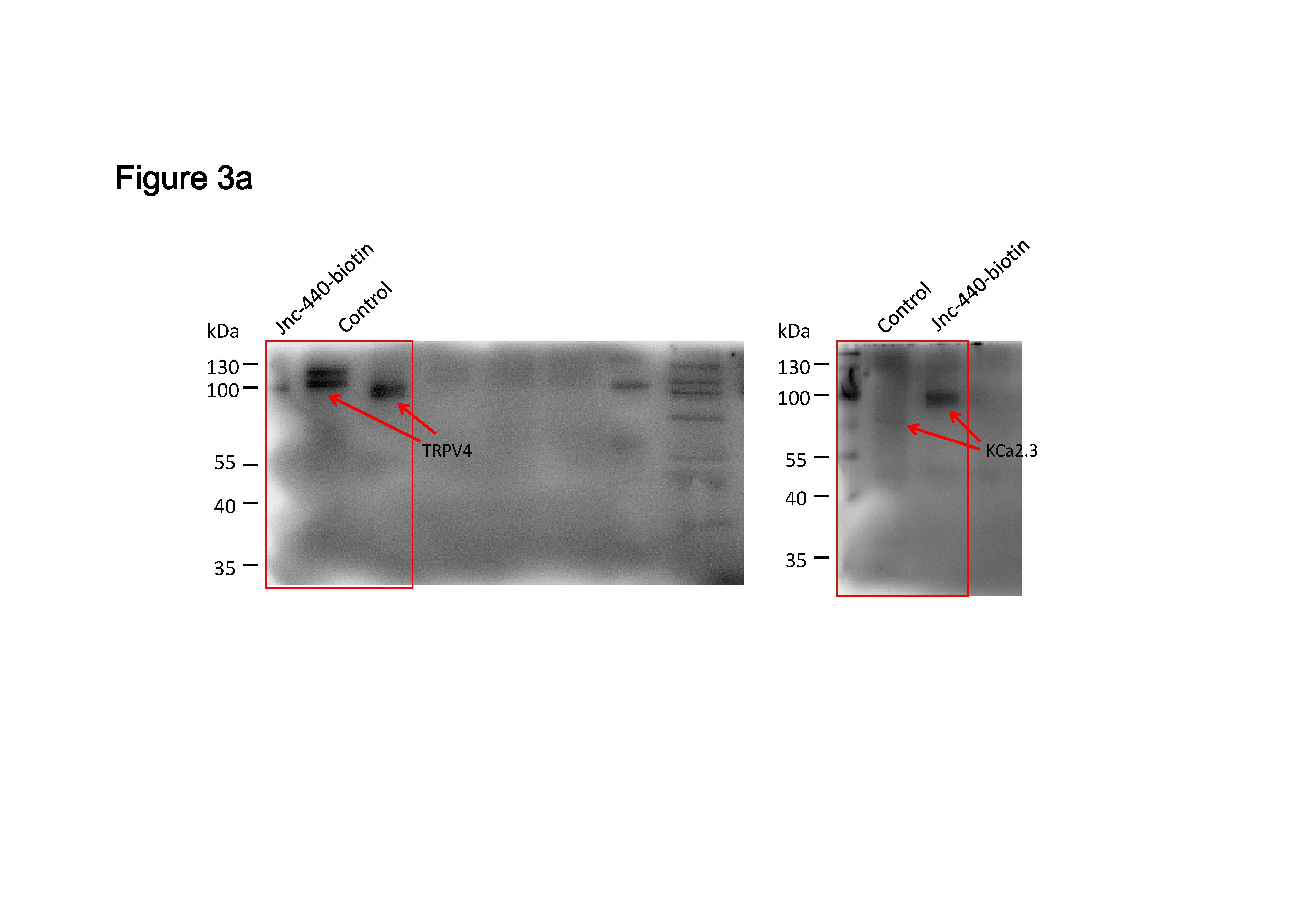

Supplement: Supplementary file 3 — Source Data for Figure 3A [file EMMM-9-1491-s002.jpg]
